# [Retracted] Functional characterization of the nitrogen permease regulator‑like‑2 candidate tumor suppressor gene in colorectal cancer cell lines

**DOI:** 10.3892/mmr.2023.13088

**Published:** 2023-09-11

**Authors:** Ai-Yun Liu, Ming-Na Liu, Feng-Hua Pei, Jing Chen, Xin-Hong Wang, Dan Liu, Ya-Ju Du, Bing-Rong Liu

Mol Med Rep 12: 3487–3493, 2015; DOI: 10.3892/mmr.2015.3881

Following the publication of this paper, it was drawn to the Editor's attention by a concerned reader that certain of the microscopic images shown in Fig. 1C on p. 3489 and the invasion assay images shown in Fig. 5 on p. 3491 were strikingly similar to data appearing in different form in other articles written by different authors at different research institutes. Moreover, unexpected similarities were identified comparing between a pair of the flow cytometric assay data panels in Fig. 4 on p. 3490, considering that these data were intended to show the results from differently performed experiments.

Owing to the fact that the contentious data in the above article had already been published, or were already under consideration for publication, prior to its submission to *Molecular Medicine Reports*, the Editor has decided that this paper should be retracted from the Journal. The authors were asked for an explanation to account for these concerns, but the Editorial Office did not receive a reply. The Editor apologizes to the readership for any inconvenience caused.

